# Water transport in subducting plate

**DOI:** 10.1093/nsr/nwaf180

**Published:** 2025-05-09

**Authors:** Yong-Fei Zheng

**Affiliations:** State Key Laboratory of Lithospheric and Environmental Coevolution, School of Earth and Space Sciences, University of Science and Technology of China, China

Plate subduction can carry water in both hydrous and nominally anhydrous minerals into different mantle depths. In this journey, water is present in the form of molecular water and structural hydroxyl in slab minerals [1]. While water in subducting slabs is predominated by hydrous minerals at lithospheric depths, nominally anhydrous minerals play a greater and greater role in the storage of water at asthenospheric depths [2]. With subduction from shallow lithosphere to deep asthenosphere, water in subducting slabs is progressively released to the overlying mantle wedge on one hand, and progressively sequestered into both hydrous and nominally anhydrous minerals at elevated temperatures and pressures on the other hand [1,3].

The proportion of nominally anhydrous minerals in subducting slabs changes with increasing both temperature and pressure [2–4]. Basaltic crust is usually metamorphosed at low geothermal gradients, giving rise to eclogite that is characterized by the occurrence of omphacitic pyroxene and garnet. Peridotitic mantle stays mineralogically stable during subduction, suffering variable degrees of reworking for the mineral assemblages of olivine, pyroxene and garnet. With respect to the storage of water in slab minerals, pyroxene has a much greater capacity than both garnet and olivine at mantle depths. Although the flux of water in slab minerals is primarily dictated by the budget of water in these minerals, partitioning of water between coexisting minerals plays a critical role in the storage of water in individual minerals. For this reason, it is intriguing whether any minerals except pyroxene can store more water in the subducting slab at depths from the top upper mantle through the transition zone to the top lower mantle.

Two sets of new experimental results are reported by almost the same group of authors for the storage of water in nominally anhydrous minerals [5,6], providing new insights into the storage and partitioning of water at mantle depths. The first set of experiments were performed in the garnet-water system under free-water-present conditions of 5–15 GPa and 827–1627°C [5], and found that the solubility of water in pyrope garnet increases with both temperature and pressure, achieving the maximum at 2000 ppm (wt. H_2_O) at the topmost lower mantle. The second set of experiments were made in the MgO-Al_2_O_3_-SiO_2_-Mg(OH)_2_ system under free-water-absent conditions of 23–32 GPa and 1327–1827°C [6], and found that the storage of water in stishovite increases almost linearly from ∼296 to ∼3611 ppm (wt. H_2_O) with increasing Al_2_O_3_ content from ∼0.3 to 3.0 wt%, but water is almost absent in coexisting bridgmanite and periclase at the top lower mantle.

It is known that the solubility of water in minerals provides a proxy for the maximum storage capacity of water in the minerals [2]. In the subducting slabs, water is present in hydrous and nominally anhydrous minerals [6] rather than occurring as an aqueous solution, as it does in the free-water-present experiments [5]. Furthermore, water is preferentially partitioned into stishovite over coexisting bridgmanite and periclase at the top lower mantle [6]. If this result is extended to the partitioning and storage of water at shallower mantle depths, the partitioning of water between coexisting minerals is a key to the storage of water in the constituent minerals. In the mineral assemblages of either eclogite or peridotite, it is possible for water to be preferentially partitioned into pyroxene rather than either garnet or stishovite. Once the two sets of new experimental results [5,6] are inspected together, they complement each other in interpreting the storage and partitioning of water in nominally anhydrous minerals. As such, the transport capacity of water in subducting slabs is primarily dictated by the budget of water in the slab constituent minerals at mantle depths. Moreover, the total amount of water in slab minerals is progressively reduced with prograde subduction from the shallow to deep mantles.
